# Loneliness in Young Adult Workers

**DOI:** 10.3390/ijerph192114462

**Published:** 2022-11-04

**Authors:** Sarah L. Wright, Anthony G. Silard

**Affiliations:** 1 Department of Management, Marketing & Entrepreneurship, University of Canterbury, Christchurch 8041, New Zealand; 2 Center for Sustainable Leadership, Luiss Business School, 00162 Rome, Italy

**Keywords:** loneliness, social relationships at work, belongingness, young adult workers, social disconnection, work practices

## Abstract

Loneliness is commonly associated with older people with the majority of research and interventions focusing on loneliness in aged and aging populations. However, loneliness seems to be on the rise for young adults more so than the elderly. Our research focusses on the experiences of young workers who report feeling lonely at work. We explore individual and organisational factors that may be contributing to loneliness, and comment on the consequences of feeling lonely at work. Qualitative data from 37 young adults from Western Europe suggest that these workers feel invisible at work, have a thwarted sense of belonging to their employing organisation, and often experience relational deficiencies due to automation and individualisation of work practices.

## 1. Introduction

*“I’m seen as this figure, in this role, but not as me … it’s really isolating being treated as a nothing, like you as a person don’t exist”* Retail worker, 23 years

*“I think it’s a time in life that often gets overlooked. I think school children tend to get a lot of interventions and things but once you leave school and go out into the big wide world you kind of get thrown out, and it’s a time of a lot of change when you’re moving away or starting a new job or going to uni, and it’s just so many changes and it can be very overwhelming and very lonely.”* Marketing assistant, 24 years

Loneliness has historically been known to predominately afflict older people, with the majority of research and interventions focussing on loneliness in aged and aging populations. Indeed, there is a vast research literature examining the prevalence and negative outcomes of loneliness in older age [1]. However, loneliness seems to be on the rise for young adults more so than the elderly. Recent research indicates that people over 65 are now more likely than any other age group to say they *never* feel lonely, with young adults (18–25 years) across Europe [2], New Zealand [3], Australia [4], the UK [5] and USA indicating higher incidences of loneliness than their older counterparts [6]. According to a recent Cigna survey of more than 6000 workers, loneliness at work is also on the rise, but it appears to be most prevalent among younger workers (<38 years), of whom nearly half report feeling lonely when they are at work [7]. In the workplace, lonely workers tend to have lower performance ratings, are less committed and less approachable than their non-lonely co-workers [8], and take twice as much sick leave [9]. The silence of loneliness and the stigma associated with it adds to the complexity of addressing these adverse outcomes.

In view of the emerging evidence of the growing prevalence of loneliness among young adults, it is helpful to gain insight into the experience of loneliness among young workers. The current study offers a preliminary understanding of loneliness among young adult workers in Western Europe by exploring the interaction between life stage as an individual characteristic and the organisational context as a socio-environmental characteristic. Such insight will ultimately lead us to develop further research studies and design age-appropriate interventions.

Loneliness is the psychological pain of perceived relationship deficiencies [10]. Perception is critical to this definition: people can live relatively solitary lives and not feel lonely, or can have many social relationships and nevertheless feel lonely. Consequently, loneliness is more closely related to the perceived quality than the quantity of social relationships and can be devastating for one’s psychological and physical health [11], even more so than obesity [12]. Loneliness is also a potent risk factor for suicide [13]; a link that is particularly evident for teenagers and young adults [14]. Feeling lonely can be particularly acute during young adulthood because this stage in life also presents the greatest risk period for the emergence of depression and magnifies the stigma of loneliness given the strong pressure to appear socially connected [15].

In addition, while the pernicious effects of rejection are felt by all age groups [16] and can cause an individual to feel like life is less meaningful [17]—even if the rejection is by a group the individual does not wish to belong to [18]—these effects can be particularly severe for young adults [19]. As such, young adults are often at greater risk for experiencing loneliness because rapid social changes are often occurring, existing support networks can be unstable, and new stressors are introduced, such as starting work and carving out an occupational or professional identity. Given this context, it seems that young adulthood is a vulnerable time for loneliness.

Importantly for the current research, young adulthood represents an era where the individual strives to form and maintain social bonds and meaningful relationships with non-family members, and explores independence and multiple facets of their potential occupational identity. We know from prior research that having social support and a sense of belonging during emerging adulthood is an important foundation for positive physical and mental health [20]. As such, studying the transition and exploration during emerging adulthood are not new avenues to explore in loneliness research. However, what is novel is the contemporary labour market young adults enter and the individual variation of their experiences. The psychology of working framework [21] is a theoretical model that can help frame the sociocultural aspects of contemporary work experiences and shape how organisational contexts can be a major influence on an individual’s psychological wellbeing. The core assumptions of the psychology of working model are that work (i) has a major influence on well-being, (ii) is intertwined with other life spheres, (iii) is shaped by socioeconomic, political, and historical factors, (iv) encompasses both paid and unpaid activities, (v) is important for workers and nonworkers who want to work, and (vi) can potentially satisfy fundamental human needs. The current study adopts these assumptions, with special emphasis on the role of work as a vehicle that shapes relational experiences.

Young adult workers are entering the workforce amidst new ways of working—in part driven by the pandemic [22]—which may be contributing to loneliness in this age group. Contemporary ways of working are also likely to see the continuing rise of digitalization, automation and individuation in young adult working lives that can increase social isolation (e.g., remote work, virtual work, piece-rate and gig-economy jobs, platform work). These changes in the way work is conducted is accompanied by changes in employment contracts to accommodate more flexible working arrangements (e.g., causal work, temporary/fixed-term/variable contracts, or self-employment). Such work often comprises a lot of time spent alone socially distant from coworkers or in temporary employment rather than in socially connected workplaces, and may increase feelings of disconnection and loneliness. Additionally, the fragmentation of the traditional ‘9-to-5’ workday shared with coworkers in-person and the rise of virtual and precarious work in the ‘gig’ economy [23] often mean a lot of time spent alone with less meaningful face-to-face interactions that might thwart the environment emerging adults need to build fulfilling social bonds. Researching the psychosocial consequences of this evolution of work is important, including studying feelings of loneliness that emerges while working. We know much about the personal factors that predispose people to loneliness (especially in childhood and older adulthood) but the interpersonal and contextual factors are much less understood, making targeted evidence-driven interventions and evaluation problematic.

Technology and social media are often touted as both the blame and remedy for youth loneliness. However, there is growing recognition that the underpinnings of loneliness are more complex and interact with environmental factors in multifaceted ways. Available evidence suggests that different age groups experience loneliness differentially in various contexts [24], and can arise from psychosocial tasks and relationships unique to that period. This evidence suggests that it is important to study life stage *and* context when understanding loneliness in the workplace. However, context is not often considered a property of loneliness, and the workplace context is given even less consideration when understanding the nature of the experience. Although data from online and media surveys [25] indicate the majority of workers feel lonely and 53% would give up some compensation for more meaningful relationships with colleagues [26], very little academic research explores how the nature of contemporary labour is contributing to loneliness. Because of the stigma and feelings of personal failure associated with loneliness, not all young adults will seek help. The stigma and associated inhibition of disclosing feelings of loneliness may be highlighted in a social context such as the workplace where interactions are often coupled with power and status differentials, which are often not in the young adult’s control or influence. In view of the increasing prevalence of loneliness among young adults in general, it is important to gain a better understanding of work and its intersection with loneliness that is unique to this developmental period [27]. Our primary research objective is to understand the experiences and consequences of loneliness at work for young adults. Our more speculative goal is to generate an awareness that loneliness is a political, economic, and social reality, rather than simply an individual problem.

## 2. Method

### 2.1. Research Design

Existing research on loneliness often associates the experience with a mental illness such as depression or social anxiety and is therefore perceived as an individual deficit. Loneliness is rarely studied from the perspective of those whom are often in the company of others, e.g., in a collective workplace (physical or virtual). Therefore, this preliminary study seeks to explore the meaning of this particular aspect of loneliness in organisational contexts. Because human existence in organisations is fundamentally interpersonal and exists in a push and pull of intersubjectivity, we used qualitative methods to explore the meaning of young worker loneliness and to begin to understand the role organisations (systems, processes, and the people that engage in them), play in its development and maintenance.

Some time ago, Rook [28] argued that researchers should take a differentiated view of loneliness and incorporate these distinctions into research methodologies. However, it is evident that most research on workplace loneliness is quantitative (e.g., [8,29,30,31,32,33,34]). We chose a qualitative inductive research design given the exploratory nature of the study and our interest in the participants’ experiences of loneliness. This approach helped us respond to our research objective of understanding the experience of participants rather than using standardised measures of loneliness to determine hypothesized predictions. We felt this approach would allow us to more fully understand the reasons why young workers identify as lonely, as the feelings of loneliness may differ widely depending on the experiences of the young worker and have various consequences.

### 2.2. Procedure

We used the data collection service provider, Prolific, to recruit participants aged 18–25 years who were employed at least part-time in a work environment interacting with other people. Prolific was used for several reasons: (i) because we wanted to pre-screen participants for their age, employment status, and degree of loneliness before they participated in the qualitative study (i.e., workers were screened for and excluded from participating if they were older than 25 years, were unemployed, and responded ’never’ or ‘hardly ever’ to the question how often they felt lonely at work); (ii) participants could remain completely anonymous using only their Prolific ID as an identifier, (iii) the questions required responses of a private and sensitive nature and thus we wanted separation from any particular sponsoring or employing organisation, and (iv) the Prolific pool of participants has shown to be of a good quality [35]. The survey was open for seven days in November 2021. Participants could choose where and on what device to complete the survey so their responses were private without fear of organizational monitoring. From a possible 40 respondents, we analysed 37 useable responses. Participants were from Portugal, Spain, Italy, England, and Poland; 14 were female, 22 were male, 1 was non-binary, with an average age of 22 years. Each participant was offered £10 to participate in the study. Informed consent was obtained prior to participation, and no information on participant identity was collected at any time. The study was reviewed and approved by the University of Canterbury Human Ethics Committee (HEC 2020/113).

We used Qualtrics to create an online anonymous survey and to collect data on participant’s experiences of loneliness in their work. Anonymous online surveys are a suitable medium to collect data on sensitive topics such as loneliness [36], and allow for the disclosure of potentially negative depictions of the respondent’s workplace, supervisor, or coworkers because of the assured anonymity. We also felt interviews with a stranger on a highly sensitive and stigmatised subject matter such as loneliness may result in socially desirable responses.

In the online survey, we provided information on the nature of the study and sought informed consent before proceeding. Participants could opt-out at any time during the survey. We asked several warm-up questions about the nature of the participant’s role, and then the following study-specific open-ended questions to elicit as much detail as possible. Example questions include: *I want you to think about a time when you have felt especially lonely at work. Tell me about that time [where were you working at the time, what tasks were you doing, who else (if anyone) were you working with, why do you think it was an especially lonely time?]. What do you do when you feel lonely at work? How does loneliness affect you? What do you think are the reasons for your loneliness?*

### 2.3. Data Analysis

All of the analysis was carried out using the verbatim text from the survey data. The length of the entries varied depending on the stories and scenarios described by the participants. Word count averaged 588 words for each participant and each took on average 46 min to complete the survey. Thematic analysis was carried out based on the procedure described by Braun and Clarke [37]. To summarise, we (i) read the text several times to build familiarity with the data, both across the entries and within each participant’s responses; (ii) created a set of broad themes, (iii) reviewed the themes for meaning and succinctness, and (iv) described the final three themes with exemplar anonymised quotes to support them. The initial coding and identification of themes was repeated by a doctoral student (not involved in the data collection) who was blind to the initial analysis. This process helped strengthen the reliability of the analysis. The labels and description of the final themes were discussed between the researchers and agreed upon after multiple rounds of discussion and analysis. Quotes reported in this paper are verbatim without grammatical correction.

## 3. Results and Discussion

Thematic analysis of the data from lonely workers suggested three main themes, which we labelled ‘feeling unheard and unseen at work’, ‘thwarted belongingness’, and ‘individuation of work’.

### 3.1. Theme 1: Feeling Unheard and Unseen at Work

*“I’d like to be of use at work, and be comfortable enough that someone higher up would want to speak with me and, like, see you, and you’re sort of actually having those conversations go in different directions and then realise that others at work don’t really listen to you”*—Oil data analyst, 23 years

Visibility at work can be thought of as the degree to which an employee is “fully regarded and recognised by others” [38] (p. 63). Several participants wrote about work environments where they did not feel they mattered, that they experienced little care or positive attention, or felt directly undermined. Their invisibility was noticeable and distressing. The experiences typically reflected two phenomena; one relating to perceived deficiencies in the participant’s character resulting in them feeling invisible or unnoticed (i.e., something is wrong with me), and another of other organizational members in the environment excluding them (i.e., no one notices me).

*“When I feel really lonely at work … you kind of think there’s something wrong with you and you think you’re lonely because no one likes you and because you don’t matter and no one cares”* Cook, 20 years

*“There are certain times when, for instance, a certain thing must be done that can be “more difficult”, so [my supervisor] decides to ask my [coworker] about it instead of me, or for him to do my work instead of me, while choosing to ignore me and the things I pitch in, only because “I’m the new one”. That feels odd, like I go unnoticed, and besides, it also feels like she [the supervisor] never trusts me”* Accounting assistant, 22 years

Dispersed through the participant’s entries were elements of a lack of care in not being seen or heard, feeling misunderstood or that the person does not matter to anyone at work.

*“no one really talks or interacts with me and I felt really lonely and unwanted”* Warehouse worker, 22 years

*“If I call in sick, no one would bother wondering what’s wrong …and then I’m blasted when I go back to work for taking sick … no one cares”* Check-in assistant, 23 years

*“… I lost a family member and was upset [at work], but I knew not to tell any of my coworkers. I felt lonely because nobody knew and nobody cared and I really wanted someone with who I could share my problems”* Salesperson, 25 years

A useful framework to help explain these observations is Buber’s [39] ‘I and Thou’ theory, which describes how people treat each other and how they learn to interact with others. Buber distinguishes between seeing people as I–It where people are used as a goal or tool toward an outcome, or I–Thou where the other is acknowledged and treated as a meaningful human being. It appears from our data that many lonely workers are in environments where people are treated as a means to an end.

When prompted to think about their loneliest experience, one participant commented that the worst aspect of that experience was

*“not being able to express myself, my feelings, or raise issues … I felt completely ignored”* [in relation to a team decision that affected their work] Graphics creator, 24 years

Another commented of the inability to talk with others about her feelings:

*“… nobody even notices you need help with [tasks], so it kind of hit me really hard that nobody even listens or even notices. I felt really lonely because it felt like there is some kind of magical barrier between us and we couldn’t really even talk about it”* Recruitment coordinator, 23 years

Jung [40] argued long ago that being lonely is not merely social isolation, but rather it includes not being heard or understood: “Loneliness does not come from having no people about one, but from being unable to communicate the things that seem important to oneself, or from holding views which others find inadmissible” (p. 356). This insight seems to resonate for many young adults in the contemporary workforce, in that they feel ‘overlooked’, ‘ignored’, or ‘silenced’. Furthermore, it was evident in the data that employees were not strategically managing their level of visibility by hiding parts of who they are, which may be a strategy exercised in more senior roles to establish and maintain professional distance [38].

In reporting the experiences of being unseen or unheard in the experience of loneliness, we are cognisant that lonely people tend to have a cognitive bias and heightened awareness of social threat [41]. Therefore, our sample of lonely workers is likely to experience and report some social interactions as more threatening or abrasive than non-lonely workers. Respondents wrote about a lack of felt care from others in the organisation, or feeling undervalued or unappreciated. It is unclear from our data whether this lack of felt care stems from a lack of reciprocity (i.e., lonely individuals are less able to capitalise on the benefits of interpersonal interaction due to heightened levels of social rejection) or the withdrawal from others that isolates them further and results in their failing to pick up on caring cues. The sad irony, though, of not having a voice or feeling invisible at work is that young lonely workers may not gain the experience of interpersonal and group interactions that might foster social skill development as they mature in their working lives. As a result, they may develop less skill in appropriately disclosing their experiences of relational deficiency, further reinforcing their distress and heightened sense of social threat. A consequence of this vicious cycle [42] is that young workers may ultimately miss the opportunity to build trusting and fulfilling relationships at work.

### 3.2. Theme 2: Thwarted Belongingness

This theme describes participants feeling disconnected from the social fabric of the organisation, which is both elemental to their loneliness and a consequence from it. Frequently, this was simply a result of age or other individual differences leading to participants feeling alienated from the in-group and not ‘fitting-in’.

*“ … here’s what I believe: Most of the times, it is as though I feel like an odd one out, like I can hardly relate to others in terms of what I’m currently experiencing: I’m the youngest at work, and my coworkers can be a bit hard to confide in, at times, so it feels like I don’t have that many people to talk to”* Accounting assistant, 22 years

*Since I was the latest “addition” to the office sometimes I feel left out with my coworkers since they have been working together for many many years and I just arrived like a few months ago, they have their own inside jokes and besides since they are men and I’m the only woman in the office it’s kinda hard for me to catch up whilst talking whenever we get to be together. At those times when they are talking/sharing experiences or memories is when i feel alone”* Administrative worker, 24 years

Not surprisingly for a sample of lonely workers, most of the participants reported various degrees of feeling only superficially connected to others at work, or being directly ostracised from the social fabric of the organisation.

*“At that moment everyone stopped talking and laughing and stared at me like if I had to say something wrong. I immediately shut up and one of the girls said “what are you laughing about? This is a personal joke, you don’t even know him”. And I just answered “Oh, I do know him from X” and then I left. I felt like the biggest idiot on earth and went to the bathroom to cry a little. I was so humiliated and ashamed that it took me a couple of minutes to recompose myself. No one ever mentioned anything about that moment again”* Hospitality frontline worker, 23 years

*“we had a hard morning and by 3 p.m we was working yet without eat. We had a break in work and we had to choose how to eat without leave the service alone. all my co-workers choose eat together and leave me alone in service. I felt that no one likes my company and that’s why they choose me to be alone”* Nurse intern, 25 years

*“I feel lonely whenever I hear them [coworkers] laugh outside my office, it makes me wish I could have someone to relate”* Polygraph processer, 24 years

*“I can get a bit desperate for connection with others”* Postal worker, 22 years

Social isolation is in itself a neutral experience. It is the affective component, the ‘*desperation*’ and ‘*wish*’ for connection that is part of the experience of loneliness, coupled with the distress of social disconnection. For this reason, loneliness can be considered a subjective rather than objective sense of social isolation. Thwarted belongingness is a “psychologically painful mental state that results when the fundamental need for connectedness is unmet” [43] (p. 2). This state is evident with many of our lonely workers. The cognitive and affective effort to process, interpret and understand the implications of being social excluded [44] (p. 841) can inhibit self-regulation of socially accepted behaviours [45] and can manifest as being ostracised from the group. As mentioned earlier, these effects of ostracism can be especially painful for young adults. This is seen in the above quote where the nurse intern realises that there must be something about their behaviour which is creating social exclusion.

For some participants, there are tentative links between workplace incivility, ostracism and thwarted belongingness.

*“There was one time when I accidently dropped a customer’s item and we couldn’t find it (it was a microSD card) and it was really stressful for me, especially because the customer was starting to get angry and stress out and my coworkers were pressuring me a lot. I eventually found it but I felt really bad about it and my coworkers didn’t communicate with me for the rest of the day”* Retail worker, 20 years

Without a sense of belonging to the group or workplace, there is no protective buffer for the young worker between the experience of incivility or the ‘silent treatment’ and feelings of ostracism. In fact, a quarter of participants used the words “anger” or “angry” in expressing narratives about their interactions with other coworkers or superiors, and corresponding with the first theme many participants conflated this hostile or ostracising behaviour with something being “wrong” with them. Such treatment or negative affective experiences can result in feelings of exclusion from the social milieu of the organisation.

According to Hagerty et al. [46] there are three main individual antecedents in developing a sense of belonging: the potential and desire for meaningful involvement, the potential for shared characteristics, and energy for involvement. Our data shows some evidence of the first two factors being thwarted in young workers’ experiences (i.e., the desperation and wish for social connection, and the feelings of not fitting in due to age or some other perceived personal deficiencies). Energy for involvement is a code that emerged in the data both from the participant’s perspective and from those they work with. That is, there is a bidirectional element—a lack of effort by others at work to connect with the referent when they are lonely coupled with a lack of effort on the referent’s behalf to connect with others.

*“When I feel really alone at work, I try to put on some wireless headphones at work and listen to some music at a low level, I usually go to the bathroom a little more often to clear up my feelings of loneliness”* Administrative worker, 24 years

*“I’m the new one, so I do feel lonely most of the time, because noone talks to me and when that happens I feel bad and decide to distract myself with my cellphone”* Check-in assistant, 23 years

*“Often I want to talk to someone and then I just go “Nooo they don’t really care, I shouldn’t bother them” and I just don’t”.* Recruitment coordinator, 23 years

As per the last two comments, there seems to be a reward and punishment mechanism built into social interaction that derails a sense of belonging. That is, social interaction is not rewarding for the individual, therefore they gravitate toward solitary engagement or acknowledge mechanisms in place whereby people are inhibited from interacting:

*“I felt more loneliness when I have my break lunch, it’s really annoying that our business has different rest times for each one. I eat total alone, sometimes is okay but the rest of the time I felt it’s not fair. They are breaking our labor relation, in my opinion”* Dental assistant, 24 years

*“Having social relationship at work is really important, but my job is a bit restrictant about interacting with others (excepting phone calls)”* Telemarketer, 24 years

The regular, continuous social interaction that can often provide a rich source of reward and social learning in organisations, in these instances, is obstructed. Through their examples, several participants mentioned work practices that inhibited social connection, such as solitary break times to keep operations moving, unpredictable work schedules, and limited collaborative activities. Focussing solely on work tasks can implicitly remove the permission to interact, and therefore stymie the opportunity for social connection.

Thwarted belongingness was also encapsulated by a lack of social support felt during times of work challenges, resulting in the worker feeling alone and vulnerable.

*“At work I feel like there is so much to figure out, and who I can ask because I don’t know everything yet. I just turned up and was kind of left alone. I wasn’t sure what do to do and that felt quite lonely”* Call centre workers, 23 years

*“I would say that the most lonely I feel when there is a very stressful day or situation at work and there is nobody around I could talk to to let the steam out”* Recruitment coordinator, 23 years

*“I like to have the support of the workers when I have doubts about something I haven’t learned how to do, so if no one is available to help me, I feel lonely”* Product promoter, 22 years

Social support is a central feature in the experience of belonging, particularly in endeavours of shared activities [47]. Furthermore, our sense of support can be adversely affected by interpersonal moments that undermine belonging needs [48]. It is important to note that while a lack of social support is a strong antecedent to loneliness, it is not the experience of loneliness itself. Rather, social support represents a set of interpersonal behaviours (or perceptions of behaviours) that are distinguishable from loneliness. Our data suggest that the affective experiences derived from a lack of social support, particularly those situations that arise from needing help, contribute to a sense of thwarted belongingness, which in turn contributes to lonely feelings.

### 3.3. Theme 3: Automation and Individuation of Work

Although much of what participants discussed as their experience of loneliness was related to real and perceived social disconnection and thwarted belonging needs, once we delved deeper it became apparent that the underpinnings of work-related loneliness are more complex than simply social disconnection. This final theme was the most diverse in terms of participant experiences of their working conditions, and took some discussion to agree upon as a defined theme. The range of work experiences were varied among participants and ranged from feeling powerless or disempowered at work (in itself not unusual for young or low-level employees) through to a lack of self-determination over *how* one works, and the repetitive, menial nature of many tasks performed by participants. We focussed on the narrative around the disconnecting nature of the work itself, and how the characteristics of their tasks resulted in loneliness.

*“I have to work and concentrate on what I am doing. so I really couldn’t communicate with anyone”* IT worker, 20 years

*“My loneliness in my job depends whether or not I sort mail by hand or by machine. When sorting by hand I feel like I have more time to interact with my colleagues. The problem is that I generally sort mail by machine a lot more. When sorting by machine I don’t really have the opportunity to interact much with others because the job is too intensive in order for any socializing to happen. I therefore often feel a bit tired and lonely during my hours at work”* Postal worker, 22 years

Our analysis shows that high demands at work were not considered problematic per se, unless accompanied by a lack of control and relational disconnection. For some workers a lack of ability to form relational connections due to the intensification of individualised work was problematic and led to feelings of loneliness.

*“… we are constantly immersed in whatever we’re doing on our computers; most of what I do is digital, so there isn’t much interaction with people, which can make it especially difficult to feel as though I am part of something”* Accounting assistant, 22 years

*“I often feel lonely during work because I start working at maximum speed and in full concentration, so I do not have time to talk to other people”* Sim racer, 20 years

Our data suggest that the frequency of informal contact between coworkers can be a significant factor in the organic formation of relationships. In other words, workers often create bonds through the simple act of day-to-day chit-chat [49]. Even though many workers felt that their work did not allow for socialising due to time or task restrictions, there was also a sentiment expressed that their loneliness arose because of the lack of informal relational connections (such as chit-chat).

*“The loneliness that I have isn’t just about human contact. It’s how that contact looks. Most of workers in my job, even in my team don’t really show any emotions whatsoever. That makes me lonely, because I don’t feel like I’m talking with human beings, but mostly a robot, that goes to work, do what he need to do without trying to talk to someone, … then go home, sleep, repeat. If you want to talk to someone about your problems, they will say “I must do this;, I don’t have time to talk”. Most of the time, I feel lonely in the work, I feel lonely even in home, and work multiplies that loneliness even more”* Database administrator, 23 years

Self-determination theory suggests that feeling supported to act autonomously elicits positive wellbeing outcomes and motivation to pursue work goals [50]. However, our analysis suggests there is an experiential difference between acting autonomously at work and autonomous work—the latter of which can be relationally disengaging. Our data suggest that the automation and individuation of work may impact loneliness in a number of inter-related ways: through generating the conditions for less meaningful connections and therefore diminished sense of unity with others [51]; and through creating job tasks that require less human contact in the workplace. Where machines replace humans or where the worker is only required to interact with a machine, it can lessen the opportunities for forming connections with others that are the foundation for generating a sense of belonging [52].

*“There’s a pain and a hole, and a feeling of emptiness and an ache in the pit of my stomach … a bitterness”* Product promoter, 22 years

For many organisations, the unintended consequences associated with relational dynamics are often not considered when implementing or changing systems and processes in the workplace [53]. Research on the expectations of younger workers suggests an increased desire for meaningful work [54] and a strong desire to be seen and appreciated as an individual [55]. This desire orients itself differently in mid-life where relationships are referenced as the core element of meaning [56]. In the workplace, a lack of input into decision making in one’s job has been found to result in emotional distress and alienation [57]. This sense of exclusion from decision-making and agency over one’s job was a cascading theme throughout our data. Extant literature suggests that a meaningful work experience is typically associated with a positive attitude towards oneself and one’s role in the organisation, having a sense of identity and purpose, utilising talents and skills or at least developing them, and having some degree of fulfilling social relationships [51]. Our data suggest that when these factors are missing in young adults’ day-to-day work experiences, and the individual desires for them to be part of their working experience, loneliness can creep into their lives.

The job characteristics model focusses on job content leading to three critical psychological states necessary for emotional health at work: (a) a feeling of personal responsibility for one’s work, (b) experiencing one’s work as meaningful, and (c) having knowledge of the results of one’s performance [58]. Skill variety, task identity, task significance, feedback from the job, and autonomy all feed into the development of these psychological states. As we learn more about how work practices and job tasks affect relational dynamics, we can see an emphasis—building on a long history of organizational and leadership research that highlights the importance of workplace relationships vis à vis a strictly task orientation that dates back to the Hawthorne Studies—towards considering relationships as the bedrock of one’s job rather than the traditional focus on task structures. This emphasis warrants further research on the relational aspects of contemporary jobs and the provision of ‘‘relational architecture’’ in organisations [59]. Without forethought on how work tasks are designed in organisations that considers the relational experience, we might find increasing numbers of young workers lonely in their work.

*“There is an absence of humanity here … I spoke about it with my mum because they really knew who they worked with but I don’t see that happening for our generation”* Recruitment coordinator, 23 years

In summary, we identified three themes in our research that contributed to participants’ feelings of loneliness: feeling unheard or unseen in their work environment, experiencing a diminished sense of belonging to the organization, and automated and individualized work leading to social disconnection. Many participants experienced work environments that emphasise individual work input/output, interpersonal emotional volatility, and systems and processes that result in limited and limiting social relationships. Such alienating values can hinder the development of any kind of desired social relationships and contribute to feelings of loneliness. Because our sample were all employees who worked onsite under supervision with others in an organisational setting, the sense of hopelessness felt in some of the participants’ narratives cannot be attributed to the often alienating working conditions associated with precarious ‘gig’ or contractor-based work [60].

## 4. Conclusions

The aim of this research was to gain some insight into the experiences and consequences of young adult workers who are lonely at work. Traditionally, the opportunity for workplace social relationships could provide companionship for individuals who may not find it elsewhere. Although work is largely a social institution, our data support the notion that merely being in a social environment is not sufficient to conquer feelings of loneliness. Although the sample is small, we have identified some of the distinctive work-related challenges and conditions that contribute to work loneliness in this cohort. Our data goes some way to support the process model of loneliness [10], in that the distress of work-related loneliness is created through a relational deficiency and the deficiency stems from individual and contextual components. Our research reinforces the notion that workers are not immune to loneliness, and supports other research indicating that workers showed more psychological distress compared to the elderly during the COVID-19 pandemic and its associated isolation requirements [61].

It is tempting to place the burden of socialising and relational fulfilment on the individuals and consider how the personal characteristics of the individual may be inhibiting the quality of their social relationships. However, given the rise of loneliness in young adults it is important to consider the ways in which the work environment operates on the individual, either causing or perpetuating loneliness. Our data support the notion that loneliness at work is not simply a personal failure, but rather can be understood as a consequence of individual, social, organisational, and economic circumstances often outside of the individual’s control. The nature of the work, and the work environment itself, might therefore be considered “loneliness-provoking factors” [62] (p. 127). An important contribution of this research is that loneliness is not simply about feeling socially isolated or lacking connection, it also encompasses not being seen, heard or understood, and feeling disconnected and marginalised from groups and institutions.

### Limitations and Future Research

This study presents a within-person analysis of the experiences of those young workers who feel lonely at work to understand what might be contributing to, and emanating from their loneliness. Future research on the relational behaviours of lonely versus non-lonely young adult workers would make for an interesting study and extend some of the ideas presented in this research on the social cognition of emerging adults in the workplace. Additionally, all of the respondents in this study were articulate in English, technically literate, and comfortable with detailing their varied experiences through a text medium. Expanding the sample would help develop the emerging themes identified in this paper. Conducting face-to-face interviews might allow for deeper exploration of participant’s experiences, which we could not explore with an anonymous survey.

Future research should increase the breadth and depth of the qualitative study with a greater number of employees from diverse organisational contexts and cultures. The participants in this study are from European countries. However, the antecedents and experiences of loneliness differ cross-culturally. For example, deficiencies with personal confidants is a stronger predictor of loneliness in individualistic societies [63], whereas lack of interactions with family are a strong predictor of loneliness in collectivist societies [64]. Future research could help understand the extent to which cultural interdependence affects the experiences of loneliness in organisations. Studying the effects of interventions is also a valuable avenue for future research: e.g., addressing maladaptive social cognitions (which has the strongest evidence of effectiveness on general loneliness; [65]; developing socialisation/on boarding processes that focus on relational aspects of work; increasing opportunities for social contact; and increasing social support.

We could end this paper on a depressing (but realistic) note about the state of loneliness in young workers, but we choose to offer an alternative view. Evidenced in the participant’s quotes is the extraordinary power and possibility of harnessing social connection within organisations to improve young workers experiences of their work and their work place/space. Individuals would not experience loneliness if they did not yearn for fulfilling social connections. Simple shifts in work practices or the way we interact with others can make meaningful differences to our experiences of work.

## Data Availability

Not applicable.
